# A Large Benign Solitary Fibrous Tumor in the Pelvis: A Unique Group

**DOI:** 10.7759/cureus.18164

**Published:** 2021-09-21

**Authors:** Karen Lei, Thiru Rajagopal

**Affiliations:** 1 General Surgery, California Northstate University, College of Medicine, Elk Grove, USA; 2 General Surgery, Mercy General Hospital, Sacramento, USA

**Keywords:** spindle cell sarcoma, surgical oncology, genetic testing, risk stratification, sarcoma, solitary fibrous tumor

## Abstract

Solitary fibrous tumors (SFTs) are a rare type of sarcoma and ubiquitous in nature, occurring anywhere in the body. Although only a few hundred cases have been described so far, certain histological features, such as hypercellularity and high mitotic index, have been associated with a more malignant course. Tumor sizes larger than 10 cm have also been associated with higher recurrence rates. There are clinical recommendations for two distinct patient groups, those with small and benign SFTs or those with large and malignant SFTs. There are few that acknowledge the unique group of those with large but benign tumors. A case involving a 62-year-old man who underwent surgical resection of a large but benign solitary fibrous tumor of the pelvis is described.

## Introduction

Solitary fibrous tumors (SFTs) are a rare type of mesenchymal tumor, which not only exhibit a wide histopathological range but can also present anywhere in the body [1]. They were first described in 1931 when one publication reported five cases of SFTs in the pleura [2]. This led to the classification of these distinct tumors as mesotheliomas or submesothelial fibromas. Since then, SFTs have been reported in extra-thoracic locations, which prompted a change of the nomenclature from mesothelioma to solitary fibrous tumor [3-5].

Less than 2% of all soft tissue tumors are SFTs [6]. Of those, approximately 34% arise within the thorax, 28% within the abdomen, 16% in the extremities, and 11% in the head and neck [7]. SFTs can be further categorized by their spindle-type histopathologic features, which exist on a spectrum ranging from a fibrous hypocellular pattern to a hypercellular pattern with diffuse branching [1,3,8]. Immunohistochemistry (IHC) has allowed for even further characterization of SFTs, distinct from other sarcomas or stromal tumors. SFTs have markers positive for cluster of differentiation (CD)-34, B-cell lymphoma 2 (Bcl-2), and vimentin, while being negative for S100, actin, and keratin [8]. CD34 represents an antigen for hematopoietic stem cells, endothelial, and mesenchymal cells, which may explain why these tumors have been discovered throughout the body [9]. Recently, a fusion gene *NAB2-STAT6* and a mutated *TERT* promoter have been implicated in the pathology of solitary fibrous tumors [10-12].

A majority of solitary fibrous tumors are benign [7]. However, in an attempt to stratify risk while managing those with SFTs, certain histological findings have been associated with a more malignant course. These findings include tumors that are hypercellular, necrotic, hemorrhagic, invasive, or have greater than four mitotic figures per 10 high-powered fields [3,13]. Although histologically benign SFTs do not possess these findings, they can display malignant features. They can invade, grow up to 40 cm, and tend to recur [14]. These characteristics contribute to poorer clinical outcomes regardless of histology [15]. The heterogeneity of SFT presentations and its rarity highlight the importance of case reports in helping to characterize the tumor for prompt diagnosis and treatment. This paper describes the case of a large symptomatic pelvic solitary fibrous tumor with benign histology and its postoperative course.

## Case presentation

We describe a case of a 62-year-old man who presented with a complaint of right-sided leg swelling and right-sided hip pain and was found to have a large intra-abdominal solitary fibrous tumor. He reported having right hip pain for the last two years, which was sharp in nature with associated numbness and tingling. The pain eventually progressed to a constant lower abdominal pain. On physical examination, the abdomen was soft and non-distended, with a visible bulge over the lower abdomen. Upon palpation, a large round non-tender mass was felt below the umbilicus.

**Figure 1:**
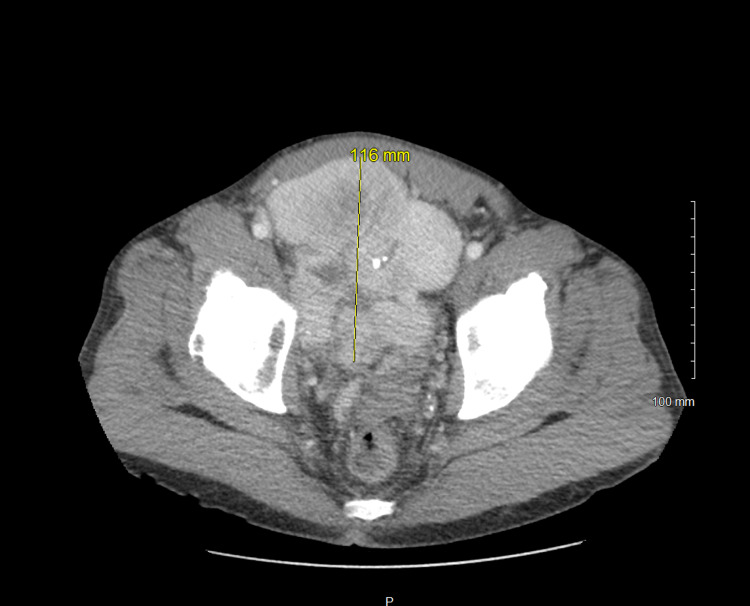
Computed tomography (CT) imaging of the abdomen and pelvis revealed a lobulated mass with heterogeneous echotexture in the anterior pelvis measuring 11.4 x 10.4 x 10.1 cm.

**Figure 2:**
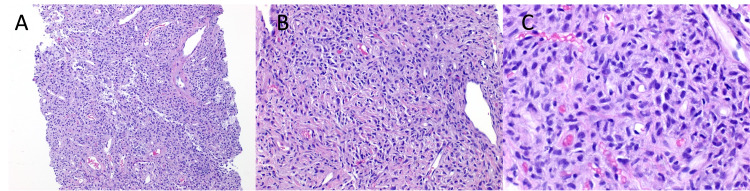
(A) 100x; (B) 200x; (C) 400x; histological slides taken from the abdominal mass biopsy at different magnifications showing a dense spindle-cell proliferation of bland spindle-cells, with vascular elements with a somewhat stag-horn morphology.

**Figure 3:**
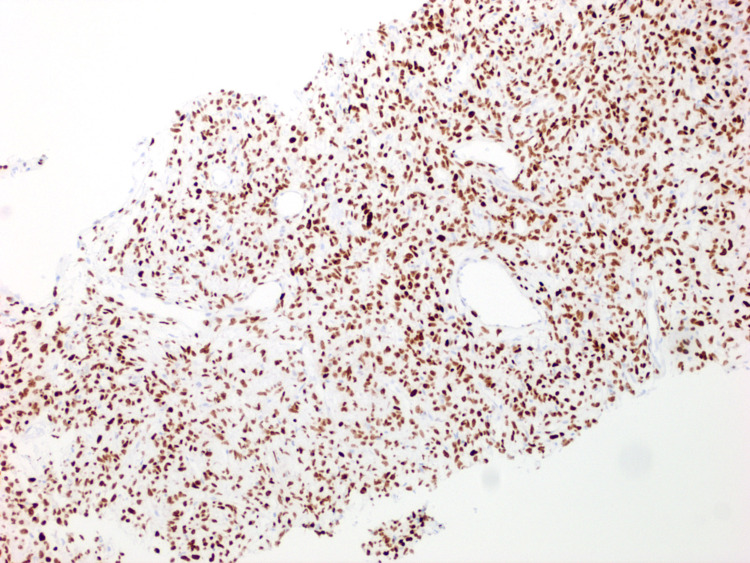
Biopsy of the abdominal mass showing positive staining for STAT6, a tumor marker for solitary fibrous tumor. STAT6: signal transducer and activator of transcription 6

Computed tomography (CT) of the abdomen and pelvis with contrast showed a lobulated and enhancing mass measuring 11.4 x 10.4 x 10.1 cm deep in the anterior pelvis with multiple flow voids (Figure 1). The mass was adjacent to the anteriosuperior surface of the prostate gland without intracapsular extension or invasion of the urinary bladder, rectosigmoid, pelvic muscles, or osseous structures. A CT-guided needle biopsy was taken, which showed a dense spindle-cell neoplasm without significant atypia or mitotic activity (Figures 2A-2C). Additionally, some sections showed cellular areas while others were hypocellular with hyalinizing features. Further immunohistochemistry (IHC) staining revealed that the tumor was positive signal transducer and activator of transcription 6 (STAT6) (Figure 3). Additionally, it stained positive for CD34 and CD99, while being negative for desmin, pan-cytokeratin (PanCK), S100, and CD117.

Three months from initial diagnosis, the patient underwent an exploratory laparotomy with resection of the pelvic tumor and cystoscopy with bilateral ureteric catheter placement. Intraoperatively, a large retroperitoneal mass arising from the posterior pubic symphysis periosteum was noted. The mass had several attachments, and its size deviated the bladder toward the left side. Due to the low-risk factor for malignant solitary fibrous tumor, the tumor was divided along the anterior surface and removed in parts. There was brisk bleeding due to the extensive tumor involvement of the pelvis, but the tumor was removed and hemostasis was secured. No gross residual tumor remained, and R1 resection was achieved. The resected mass measured 15.0 x 14.0 x 8.5 cm and weighed 448 g. The specimen was subsequently sent for histological confirmation, and the postoperative course was uncomplicated. Upon review of the tissue sections, the tumor was confirmed to be a benign solitary fibrous tumor with positive tumor marker staining and a low mitotic index. During a follow-up telephone conversation with the patient at one month post-surgical resection, the patient felt that the surgery went well and no longer endorsed abdominal pain.

## Discussion

Solitary fibrous tumors within the abdomen and pelvis are largely asymptomatic unless they grow and cause nonspecific symptoms related to mass effect [16]. These symptoms include abdominal pain, distention, constipation, urinary retention, or urinary frequency. The tumors can also invade the bladder or ureter causing hematuria [17,18]. Rarely, SFTs can occur as a paraneoplastic syndrome, manifesting as hypoglycemia due to tumor release of insulin-like growth factor-1 (IGF-1) [16,17]. These were not present in this patient. Rather, the patient complained of vague abdominal pain in the later course of the disease, suggesting pressure caused by the large abdominal tumor. Since there was no evidence of intracapsular extension into other structures, we doubt the symptoms were caused by direct invasion. Notably, the presenting complaint was of right hip pain and right leg swelling with associated numbness and tingling. Although the large tumor burden could have contributed to the chronic hip and leg pain, it is most likely secondary to degenerative changes or arthritis in the hip. The patient had multiple surgeries involving his right knee, which could have led to joint instability and pain radiating to the hip. These explanations are supported by the fact that the abdominal pain resolved, but the patient continued to have difficulty in walking following resection of the tumor. Other considerations for the symptoms include possible cerebrovascular injury as the patient reported a transient ischemic attack five months prior with no residual deficits. The patient also has prominent varicose veins, which may contribute to the leg swelling although it commonly presents bilaterally.

The patient was scheduled for follow-up appointments at two weeks and three months post en-bloc resection of the tumor, which was completed with a normal postoperative course. Repeat MRIs to assess tumor recurrence will be completed at the follow-up visits. Although the SFT in this patient was histologically benign, its large tumor size greater than 10 cm causing chronic compressive symptoms is considered a malignant feature. Currently, surgical resection with wide margins is the primary treatment of solitary fibrous tumors, with a five-year survival rate of 59-100% and a 10-year survival rate of 40-89% [16]. This is a logical solution for benign tumors as invasion of adjacent structures is uncommon and there are no distant metastases. Local recurrence in benign tumors has been reported to be up to 8%, compared to the recurrence rate of 63% in true malignant SFTs [16]. Since SFTs are rare, there are currently no guidelines on postoperative surveillance specific to this disease. Some have suggested increased monitoring in patients with tumors that are greater than 10 cm and have malignant histological characteristics, which are highly correlated with a worse prognosis [6]. This patient is unique in that he belongs to a distinct subgroup of patients exhibiting tumors larger than 10 cm but with benign histology. In one large case series involving 79 patients with SFTs, 17 similar patients with large tumors and benign histology had favorable outcomes with a median follow-up time of 18 months, suggesting that tumor size alone does not necessarily dictate prognosis [6].

Further risk stratification for this rare tumor can potentially be done with genetic testing. Several discoveries have been implicated as drivers of SFT development, including a *NAB2-STAT* fusion gene, which explains how STAT6 marker positivity is 98% sensitive for SFTs [19]. Three variants of the *NAB2-STAT* fusion gene have been described, and all have been shown to be important in tumor cell differentiation and migration but not with malignant progression. On the other hand, mutations in a *TERT* promoter or *TP53* have been associated with malignant potential in the form of necrosis, recurrence, or metastasis [20]. Although genetic testing was not done with our patient, identification of these other mutations may help in treatment planning. The genetic testing was offered as an option for this patient but was not completed. This is especially significant for patients possessing solitary fibrous tumors with an unclear disease course, those with large but histologically benign tumors and those with small but histologically malignant tumors. Patients within these subgroups may benefit from increased surveillance or more careful surgical resection of the tumor with negative margins if it is found that they carry genetic mutations associated with poor prognosis.

## Conclusions

A majority of solitary fibrous tumors are small in size and histologically benign, while a small fraction of SFTs is larger than 10 cm and histologically malignant. These characteristics aid in risk stratification and assess the need for increased surveillance. However, patients with large but benign tumors are in a unique position. Despite histologically benign features, past research has shown that a large tumor size alone is associated with increased recurrence and poor prognosis. Patients in this unique group, such as the one described in this case report, would benefit from a personalized follow-up course dependent on patient-specific tumor characteristics. Genetic testing for *NAB2-STAT* or *TERT* promoter mutations may be useful for risk stratification in these groups of patients.
